# 

**Published:** 1913-11

**Authors:** 


INDEXED

Contents, Adv. Page 5. Index Advertisements, Page 6. Publicity Dept., Page 22.

Yearly Vol. 69 NOVEMBER, 1913 No. 4

### Buffalo Medical Journal

Established 1845 by AUSTIN FLINT, M.D.

EDITOR AND PUBLISHER:

A. L. BENEDICT, A.M., M.D., 228 Summer St., Buffalo, N. Y.

ASSOCIATE EDITORS:

*NEW YORK STATE.*

Grover W. Wende, M. D., Buffalo.

Charles W. Hennington, B. S., M. D., Rochester.

Charles Haase, M. D., Elmira.

Fayette H. Peck, M. D., Utica.

Martin B. Tinker, B. S., M. D., Ithaca.

H. I. Davenport, A. M., M. D., Auburn.

*FOREIGN.*

Sir William Osler, M. D., LL. D., Oxford, Eng.

Prof. P. K. Pel, M. D., Amsterdam, Holland

Prof. C. A. Ewald, M. D., Berlin, Germany.

Rene Gaultier, M. D., Paris, France.

J. George Adami, M. D., LL. D., F. R. S. Montreal.

Henry W. Hill, LL. D., Buffalo, Associate Editor in Medical Jurisprudence.

